# A cross-sectional study on the perceived barriers to physical activity and their associations with domain-specific physical activity and sedentary behaviour

**DOI:** 10.1186/s12889-022-13431-2

**Published:** 2022-05-26

**Authors:** Yen Sin Koh, P. V. Asharani, Fiona Devi, Kumarasan Roystonn, Peizhi Wang, Janhavi Ajit Vaingankar, Edimansyah Abdin, Chee Fang Sum, Eng Sing Lee, Falk Müller-Riemenschneider, Siow Ann Chong, Mythily Subramaniam

**Affiliations:** 1 Research Division, Institute of Mental Health, Singapore, Singapore; 2 Admiralty Medical Centre, Khoo Teck Puat Hospital, Singapore, Singapore; 3 Clinical Research Unit, National Healthcare Group Polyclinics, Singapore, Singapore; 4 Lee Kong Chian School of Medicine, Nanyang Technological University, Singapore, Singapore; 5 Saw Swee Hock School of Public Health, National University of Singapore, Singapore, Singapore; 6 Yong Loo Lin School of Medicine, National University of Singapore, Singapore, Singapore

**Keywords:** Physical activity, Sedentary behaviour, Barriers to physical activity, Zero-inflated model

## Abstract

**Background:**

Physical inactivity and sedentary behaviour have detrimental consequences to the individual and the economy. Our study examined the prevalence of perceived barriers to physical activity in Singapore’s adult population and their associations with physical activity and sedentary behaviour.

**Methods:**

This cross-sectional analysis utilised data from a nationwide survey in Singapore. Participants (*n* = 2867) were recruited from February 2019 to March 2020. The independent variables were internal (e.g. fatigue, age) and external (e.g. weather, cost) perceived barriers to physical activity. The outcomes were domain-specific physical activity (work, transport and leisure) and sedentary behaviour, all of which were assessed using the Global Physical Activity Questionnaire. The associations were examined using zero-inflated negative binomial regressions for physical activity and linear regression for sedentary behaviour.

**Results:**

The median (Interquartile range) for work-related, transport-related and leisure-related physical activity were 0 (0 – 1440), 600 (160 – 1120) and 360 (0 – 1080) MET (metabolic equivalent)-minutes per week. The median sedentary behaviour (IQR) was 360 (240 – 540) minutes per day. The top three barriers were lack of time (65.3%), fatigue (64.7%) and pollution (56.1%). After adjustment, the level of transport-related physical activity was lower for respondents who cited lacking pavement or parks as a barrier, but higher for those who indicated cost and safety concerns. Respondents who reported pollution as a barrier were more likely to engage in transport-related physical activity. The level of leisure-related physical activity was lower for respondents indicating weather, lack of time and age as barriers, but higher for those reporting safety concerns. The odds of engaging in leisure-related physical activity was lower for those citing age, cost and fatigue as barriers, but higher for those indicating the weather. Sedentary behaviour was positively associated with work and limited accessibility to exercise facilities, but negatively with safety concerns.

**Conclusion:**

Individuals can be motivated to overcome internal barriers (fatigue, lack of time, cost and age) through social support and emphasis on exercise benefits. External barriers (weather and lack of pavements or parks) can be reduced by raising awareness of existing infrastructure. Sedentary behaviour can be improved by implementing workplace measures, such as reducing the time spent sitting.

**Supplementary information:**

The online version contains supplementary material available at 10.1186/s12889-022-13431-2.

## Background

According to the World Health Organisation (WHO), being physically active involves participating in moderate-intensity exercise for 150 min or vigorous-intensity exercise for 75 min per week among adults [1]. This recommendation is equivalent to 600 metabolic equivalent (MET)-minutes. However, approximately 28% of adults worldwide failed to meet this requirement in 2016 [1]. Being physically inactive poses physical health risks (e.g., obesity and chronic conditions) and increases the likelihood of mental health conditions (e.g., depression and dementia) in the individual [2–5]. Moreover, these negative health consequences can impose an economic burden at the societal level. According to a study that used data from 142 nations, the healthcare systems incurred 53.8 billion international dollars in 2013 because of physical inactivity [6].

Similar to physical inactivity, sedentary behaviour is also a concern. Sedentary behaviour encompasses any waking activity that uses little energy (≤ 1.5 metabolic equivalents (METs)) while sitting, lying, or reclining [7, 8]. Technological advancement and urbanisation have attributed to the increase in sedentary behaviour [9, 10]. The invention of smartphones and laptops has encouraged higher screen time and a greater preference for sedentary behaviour [9]. Studies have also associated sedentary behaviour with poor health outcomes, such as the increased risk of diabetes and poor quality of life [11, 12].

Perceived barriers to physical activity are features that a person views as impediments to physical activity [13]. They can be classified into internal and external barriers. Internal barriers relate to personal factors such as attitudes and preferences, while external barriers refer to the environment, such as infrastructure [14]. As populations, culture, and socio-economic settings differ between countries, barriers to physical activity can also vary. For example, an Australian-based study showed that among those who were physically inactive, the two most commonly cited barriers were lack of time (50.0%) and lack of enjoyment (43.9%) [15]. Another study in Kuwait revealed that hot weather (75.9%) and work (71.2%) were the two most reported barriers [16].

Perceived barriers to physical activity may influence both physical activity and sedentary behaviour. An Australian-based study by Salmon et al. revealed that respondents who cited cost, fatigue, and work commitment as barriers were more likely to be physically inactive [17]. Moreover, respondents who cited cost, weather, and family needs were more likely to engage in sedentary behaviour [17]. Studies related to behavioural economics have alluded to a possible explanation for the association between perceived barriers to physical activity and sedentary behaviour [18, 19]. Whether individuals prefer physical activity or sedentary behaviour is influenced by factors such as the environment and the value assigned to each activity [18, 19]. As a result, perceived barriers to physical activity may promote sedentary behaviour, as it discourages physical activity [17].

Singapore is a multi-ethnic city-state in Southeast Asia with a population that comprises 75.9% Chinese, 15.0% Malay, 7.5% Indian, and 1.6% other races [20]. The National Population Health Survey in 2019 found that the proportion of Singaporeans engaged in exercise increased from 29.4% in 2017 to 35.2% in 2019 [21]. This trend may be attributed to population-level campaigns that motivate individuals to live healthy lifestyles [22], such as the National Steps Challenge [23]. It is a seasonal campaign whereby participants are provided wearables to track their daily steps and heart rate while participating in exercises [22]. They receive financial incentives if they reach specific targets [22]. These campaigns were launched in response to counter the potential increase in Singapore’s chronic disease burden. Type 2 Diabetes Mellitus, a highly prevalent chronic condition in this multi-ethnic population, is expected to rise from 7.3% in 1990 to 15% by 2050 [24]. Another common chronic condition, hyperlipidemia, had increased from 25.2% in 2010 to 33.6% in 2017 [25].

Although several studies have examined the barriers to physical activity in Singapore, these studies only included a selected group of Singaporeans [26, 27]. Hence, the findings cannot be generalised to the Singapore population. Moreover, few studies have investigated barriers to physical activity at a population level in Singapore. The previous population-based study examining barriers to physical activity in Singapore was the National Health Surveillance Survey in 2007 [28]. The study was conducted over ten years ago. It included responses from sedentary individuals and excluded those who were physically active. Thus, our study utilised data from a representative sample of adults in Singapore to determine the prevalence of different perceived barriers to physical activity. We also examined the associations of these barriers with domain-specific physical activity (work, transport and leisure) and sedentary behaviour in the population.

## Methods

### Population

This cross-sectional secondary analysis used data from a nationwide survey that examined the knowledge, attitudes, and practices of diabetes in Singapore. The methodology was described in detail previously [29]. Participants were Singaporeans or permanent residents, ≥ 18 years, living in Singapore during the survey period and fluent in English, Malay, Chinese, or Tamil. The survey excluded individuals below 18 years old, those who could not be contacted due to missing or incomplete addresses, lived outside of Singapore, or were institutionalised during the study period. In addition, individuals who had difficulty completing the survey due to physical, mental, or cognitive impairment were also excluded. The survey was conducted face-to-face in either one of Singapore’s four main languages: English, Malay, Chinese, or Tamil. Responses were recorded via computer-assisted personal interviews (CAPI) using handheld tablets.

The recruitment period was from February 2019 to March 2020. This study excluded participants recruited between April 1st, 2020 and September 1st, 2020 (*n* = 28) because of the COVID-19 pandemic and the resultant self-imposed restriction of movement, as well as social distancing measures, implemented that may have influenced physical activity level and sedentary behaviour.

The study was approved by the Institute of Mental Health’s Institutional Research Review Committee (IRRC) and the National Healthcare Group’s Domain Specific Review Board (Ref: 2018/00430). All participants provided written informed consent. Parental consent was also obtained for participants aged 18 to 20 years old.

### Sampling and sample size

A disproportionate stratified sampling design was used to sample participants from a national administrative database that comprises of all Singapore residents. The proportion of participants in each ethnic group (Chinese, Malay, and Indian) was fixed at approximately 30%. In each age group (18 – 34 years, 35 – 49 years, 50 – 64 years, 65 years and above), the proportion of respondents was specified at 20%. This survey design ensured that the different ethnicities and age groups were well represented. Additionally, survey weights were incorporated to ensure that the sample was representative of the population.

The sample size in the survey was 3000, which was determined by the prevalence of diabetes knowledge in Singapore (60%), as reported in a previous study [30]. Moreover, it was calculated using a power of 0.8, type I error of 0.05, and adjusted for design effects. The margin of error was 2.5% for the overall prevalence and 4.5–5.0% for prevalence stratified by age groups and ethnicities.

### Recruitment strategy

The participants were sampled from a national administrative database that included all the residents in Singapore [29]. An invitation letter was sent to the participants 1 to 2 weeks before the household visit by the interviewer [29]. The letter provided information about the study and a contact number to address any enquires about the study [29]. A maximum of 10 visits were made to reduce the survey’s non-response rate [29]. If the participant was not at home during the visit, a card with the survey firm’s contact number was left in the letterbox [29]. An inconvenience fee of SGD40 was given to the participant after the survey [29].

### Barriers to physical activity

Respondents were asked to rate 12 barriers on a three-point Likert scale: ‘not really a barrier’, ‘somewhat a barrier’, ‘very much a barrier’. Internal barriers comprised ‘a disability or injury’, ‘young children or family needs’, ‘work’, ‘lack of time’, ‘age’ and ‘feeling tired’. External barriers consisted of ‘the weather’, ‘pollution’, ‘safety concerns’, ‘limited accessibility’, ‘cost of exercising’ and ‘lack of footpath, cycle lanes or parks’. An item was considered a barrier if the respondent indicated it as either ‘somewhat a barrier’ or ‘very much a barrier’. These barriers were identified through literature review and consultation with clinicians, an epidemiologist and policymakers working in the diabetes prevention domain [31–33]. Moreover, the content validity of the questionnaire was assessed through cognitive interviews. We asked the participants if they felt the items in the questionnaire represented the most important barriers and whether we had left out anything relevant.

### Physical activity and sedentary behaviour

Information on physical activity and sedentary behaviour was collected using the Global Physical Activity Questionnaire (GPAQ). It is a 16-item self-reported questionnaire that assesses two types of physical activity intensity in three different domains: moderate (work, transport and recreation) and vigorous (work and recreation) intensity [34]. Moderate intensive activities are those that involve ‘a small increase in breathing or heart rate for at least 10 min continuously’ (e.g., brisk walking), whereas vigorous intensive activities are those that involve ‘a large increase in breathing or heart rate for at least 10 min continuously’ (e.g., running) [34]. The energy expenditure for each domain was computed by multiplying the metabolic equivalent (MET) values to time variables. For moderately intensive activities, the MET value is 4. The MET value for vigorous intensive activities is 8 [35].

The physical activity level in each domain was calculated by summing the energy expenditure. Overall physical activity level was computed by adding the energy expenditure from the three domains. Sedentary behaviour was determined by asking the amount of time spent sitting or reclining per day. GPAQ was used to assess physical activity and sedentary behaviour because studies in Singapore have demonstrated that it correlated moderately with accelerometer-measured physical activity and sedentary behaviour [36, 37]. Moreover, GPAQ is inexpensive to administer in population-based studies [36].

### Confounders

Based on a previous analysis by Lau et al. [38] and two systematic reviews by O’Donoghue et al. and Bauman et al. [39, 40], we adjusted for the following confounders when examining the association between barriers to physical activity and physical activity-related outcomes: age, sex, ethnicity, monthly personal income, chronic physical conditions, and sedentary behaviour. Moreover, the systematic review by Bauman et al. found that significant environmental factors of leisure-related activity include safety concerns, access to facilities and the presence of pavement [40]. Hence, ‘safety concerns’, ‘limited accessibility’ and ‘lack of footpath, cycle lanes or parks’ were included as confounders regardless of their significance in multivariable regression analyses for leisure-related activity.

For sedentary behaviour, we adjusted for the following confounders: age, education, marital status, monthly personal income, body mass index, chronic physical conditions, and physical activity. Confounding environmental factors included ‘safety concerns’, ‘weather’, and ‘limited accessibility’. These confounders were obtained from previous analysis by Lau et al. and the systematic review by O’Donoghue et al. [38, 39].

Sociodemographic factors were included in the analysis with the following categories: age (18 – 34 years, 35 – 49 years, 50 – 64 years, 65 years and above), sex (male and female), ethnicity (Chinese, Malay, Indian, Others), educational qualification (primary or below, secondary, pre-university/junior college, vocational institute, diploma, degree and above), marital status (single, married, divorced, separated/widowed/divorced), monthly personal income (no income/below SGD 2000, SGD 2000 – 3999, SGD 4000 – 5999, SGD 6000 – 9999 and SGD10 000 and above) and WHO Classification of body mass index (Underweight, Normal weight, Overweight and Obese).

Chronic conditions were assessed using a self-reported checklist of 18 chronic conditions. These conditions included asthma, arthritis, back problems, cancer, chronic inflammatory bowel disease, chronic lung diseases, congestive heart failure, diabetes, heart disease, hyperlipidaemia, hypertension, kidney failure, migraine, neurological conditions, Parkinson’s disease, stomach ulcer, stroke, and thyroid disease. These responses were divided into three categories: no chronic condition, one chronic condition, and two or more chronic conditions.

### Statistical analysis

Survey weights were included in the analysis to account for disproportionate sampling, non-response bias, and post-stratification by age and ethnicity. Weighted percentages and unweighted frequencies were used to summarise categorical variables. The outcomes examined were domain-specific physical activity (work, transport and leisure) and sedentary behaviour.

All physical activity-related outcomes were positively skewed and had many zeros. Hence, associations between barriers to physical activity and these outcomes were determined by considering four regression models: Poisson regression, negative binomial regression, zero-inflated Poisson model and zero-inflated negative binomial model [41]. The zero-inflated negative binomial model was selected as the best model as it had the lowest Akaike Information Criteria (AIC) [41].

The zero-inflated negative binomial model is a two-part model. The first portion follows a negative binomial distribution and relates to the change in physical activity level among those who were physically active [42]. The exponential form of the regression coefficient denotes the proportional change in physical activity level (in MET-minutes) for each unit increase in the independent variable [42]. The second portion follows a logit probability process to distinguish between physically inactive respondents (represented by excessive zeros) and physically active respondents [42]. The exponential form of the regression coefficient indicates the odds of being physically inactive for each unit increase in the independent variable [42]. The exponential form of the regression coefficients (e^β^) and 95% confidence interval (e^95% CI^) were calculated for all zero-inflated negative binomial models.

Associations between the barriers and sedentary behaviour were assessed using a multivariable linear regression. Linear regression assumes that the residuals have constant variance and a normal distribution. These assumptions were tested by inspecting the residuals versus fitted values plot and the quantile–quantile plot of the residuals. These plots showed that the assumptions had not been seriously violated. Beta-coefficients (β) and 95% confidence interval (CI) were reported for sedentary behaviour.

All regression models were modelled based on the parsimony principle. Initially, all barriers to physical activity were included. Statistically insignificant barriers were removed from the multivariable model in a stepwise manner. The final multivariable model for each outcome included significant barriers to physical activity and the established confounders. Due to the disproportionate stratified sampling design, standard errors were estimated using Taylor series linearisation.

The analysis was conducted using Stata/SE 17.0 (College Station, Texas), with a two-sided test at a 5% significance level. Missing data were removed in a listwise manner.

## Results

**Fig. 1:**
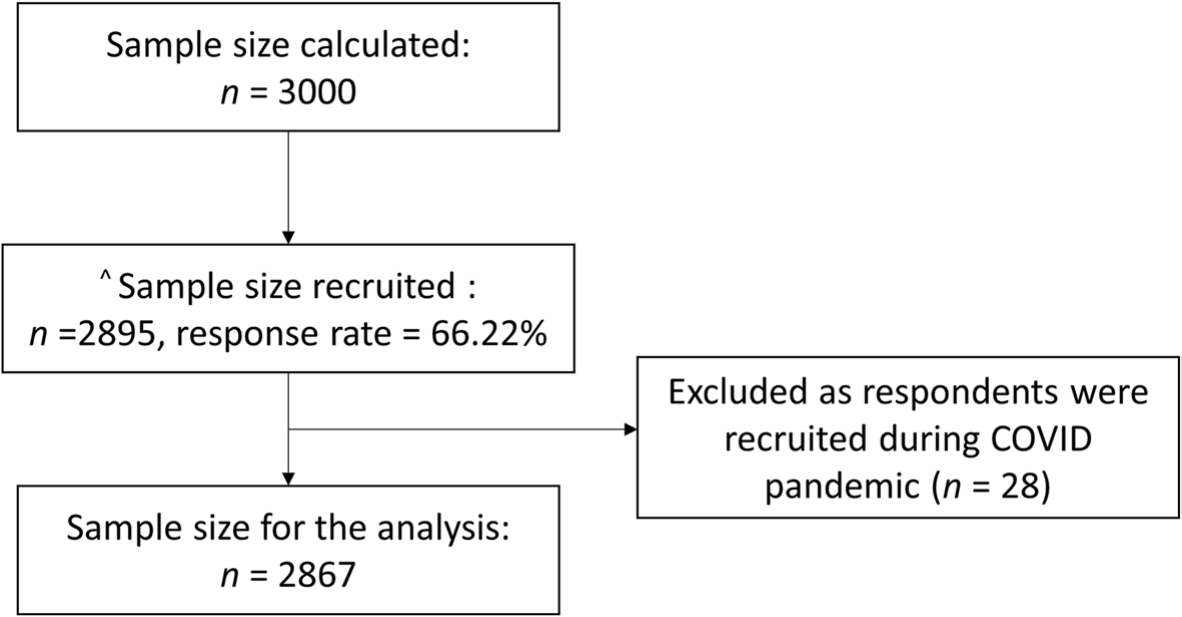
Study Flow diagram. ^^^ Sample size did not reach 3000 as the study stopped due to COVID restrictions

The survey’s response rate was 66.2%. A total of 2867 participants were included in the analysis (Fig. 1). Most respondents were 18 to 34 years old (29.9%), female (51.6%), of Chinese ethnicity (75.9%), having a university degree, professional certification or above (29.6%), married/cohabiting (61.6%), with monthly personal income below SGD 2000 (47.3%) and with no chronic condition (45.9%) (Table 1). The median (interquartile range, IQR) for work-related physical activity, transport-related physical activity and leisure-related physical activity were 0 (0 – 1440), 600 (160 – 1120) and 360 (0 – 1080) MET-minutes respectively (Table 2). Sedentary behaviour had a median (IQR) of 360 (240 – 540) min (Table 2).

**Table 1:** Summary statistics of the population (*n* = 2867)

	**Weighted Percentage (*n*)**
**Age groups (years)**	
18 to 34	29.9% (814)
35 to 49	28.2% (711)
50 to 64	26.7% (766)
65 and above	15.2% (576)
**Sex**	
Female	51.6% (1458)
Male	48.4% (1409)
**Ethnicity**	
Chinese	75.9% (791)
Malay	12.7% (961)
Indian	8.6% (908)
Others	2.9% (207)
**Education**	
Primary and below	20.4% (631)
Secondary	20.3% (681)
Pre-University/Junior College	4.7% (123)
Vocational Institute/ITE	6.6% (263)
Diploma	18.5% (474)
Degree, professional certification, and above	29.6% (695)
**Marital Status**	
Single	29.3% (723)
Married/Cohabiting	61.6% (1840)
Separated/Widowed/Divorced	9.2% (303)
**Monthly Personal income (SGD)**	
Below 2,000 /no income	47.3% (1441)
2,000 to 3,999	25.1% (689)
4,000 to 5,999	13.5% (317)
6,000 to 9,999	8.1% (180)
10,000 and above	6.0% (116)
**Number of chronic conditions**	
No chronic condition	45.9% (1216)
1 chronic condition	26.4% (759)
2 or more chronic conditions	27.7% (884)
**Body Mass Index (WHO Classification)**	
Underweight	7.3% (150)
Normal weight	55.9% (1253)
Overweight	27.4% (848)
Obese	9.4% (415)

Missing observations: marital status (*n* = 1), monthly personal income (*n* = 124), number of chronic conditions (*n* = 8), body mass index (*n* = 201)

**Table 2:** Summary statistics of physical activity and sedentary behaviour in the population (*n* = 2867)

	**Median (IQR)**
**Overall physical activity, MET-minutes**	1840 (840 – 4200)
**Work-related physical activity, MET-minutes**	0 (0 – 1440)
**Transport-related physical activity, MET-minutes**	600 (160 – 1120)
**Leisure-related physical activity, MET-minutes**	360 (0 – 1080)
**Sedentary behaviour, minutes**	360 (240 – 540)

Missing observations: overall physical activity (*n* = 1), work-related physical activity (*n* = 1), transport-related physical activity (*n* = 1), leisure-related physical activity (*n* = 1), sedentary behaviour (*n* = 1)

Table 3 presents the prevalence of perceived barriers to physical activity. The three most common perceived barriers to physical activity were lack of time (65.3%), feeling tired (64.7%), and pollution (56.1%). When the prevalence of perceived barriers to physical activity were stratified by sex, age group and race (Table S1 and S2), lack of time and feeling tired remained the top two barriers.

**Table 3:** Prevalence of perceived barriers to physical activity

	**Not really a barrier**		**Somewhat of a barrier/Very much a barrier**	
	Weighted %	*n*	Weighted %	*n*
A disability or injury	80.4	2239	19.6	624
Young children or family needs	73.7	2110	26.4	753
Work	51.9	1602	48.1	1260
The weather (e.g., wet and hot)	50.4	1599	49.6	1263
Pollution—Haze	43.9	1360	56.1	1503
Lack of time	34.7	1110	65.3	1754
Cost	78.0	2219	22.0	643
Safety concerns (e.g., street lighting, traffic)	76.8	2239	23.2	625
Limited accessibility of gym or other exercise facilities (e.g., distance hours, open, availability)	74.1	2160	25.9	685
Age	80.2	2279	19.7	584
Lack of footpaths, cycle lanes or parks	84.0	2450	16.0	414
Feeling tired	35.3	1104	64.7	1760

Missing observations: A disability or injury (*n* = 4), young children of family needs (*n* = 4), work (*n* = 5), weather (*n* = 5), pollution (*n* = 4), lack of time (*n* = 3), cost (*n* = 5), safety concerns (*n* = 3), limited accessibility (*n* = 22), age (*n* = 4), Lack of footpaths, cycle lanes or parks (*n* = 3), feeling tired (*n* = 3)

Table 4 shows the multivariable models for physical activity. No significant barriers to physical activity were found for work-related physical activity. The level of transport-related physical activity was negatively associated with a lack of footpaths, cycle lanes or parks (e^β^ = 0.79, e^95% CI^ = 0.66 – 0.94), but positively associated with cost (e^β^ = 1.33, e^95% CI^ = 1.04 – 1.69) and safety concerns (e^β^ = 1.23, e^95% CI^ = 1.01 – 1.51). Pollution was associated with a lower odds (e^β^ = 0.55, e^95% CI^ = 0.41 – 0.73) of being physically inactive in the transport domain.

**Table 4:** Adjusted models for the association between barriers to physical activity and physical activity

**Transport-related physical activity** ^**┼**^	**Physical activity level (Negative binomial model)**		**Odds of being physically inactive (Logit model)**	
Barriers to physical activity	e^β^ (e.^95% CI^)	*p*-value	e^β^ (e.^95% CI^)	*p*-value
Lack of footpaths, cycle lanes, or parks	0.79 (0.66—0.94)	0.007		
Cost	1.33 (1.04—1.69)	0.021		
Safety concerns (e.g., street lighting, traffic)	1.23 (1.01—1.51)	0.044		
Pollution – Haze			0.55 (0.41—0.73)	< 0.001
**Leisure-related physical activity **^**╪**^	**Physical activity level (Negative binomial model)**		**Odds of being physically inactive (Logit model)**	
Barriers to physical activity	e^β^ (e.^95% CI^)	*p*-value	e^β^ (e.^95% CI^)	*p*-value
The weather (e.g., wet and hot)	0.85 (0.75—0.98)	0.022	0.69 (0.53—0.91)	0.009
Lack of time	0.73 (0.62—0.86)	< 0.001		
Age	0.82 (0.67—0.99)	0.042	1.43 (1.02—2.01)	0.037
Safety concerns (e.g., street lighting, traffic)	1.29 (1.08—1.54)	0.004		
Cost			1.58 (1.13—2.22)	0.008
Feeling tired			1.85(1.40—2.46)	< 0.001

^**┼**^Adjusted for age, ethnicity, sex, personal income, chronic conditions, sedentary behaviour.

^**╪**^Adjusted for age, ethnicity, sex, personal income, chronic conditions, sedentary behaviour, safety concerns, limited accessibility and lack of footpaths, cycles lanes or parks

Weekly MET-minutes of leisure-related physical activity was lower for respondents who reported weather (e^β^ = 0.85, e^95% CI^ = 0.75 – 0.98), lack of time (e^β^ = 0.73, e^95% CI^ = 0.62 – 0.86) and age (e^β^ = 0.82, e^95% CI^ = 0.67 – 0.99) as barriers. A higher level of leisure-related physical activity was found for respondents who reported safety concerns (e^β^ = 1.29, e^95% CI^ = 1.08 – 1.54) as barriers. The weather (e^β^ = 0.69, e^95% CI^ = 0.53 – 0.91) was associated with a lower odds of being physically inactive in the leisure domain, whereas age (e^β^ = 1.43, e^95% CI^ = 1.02 – 2.01), cost (e^β^ = 1.58, e^95% CI^ = 1.13 – 2.22), and fatigue (e^β^ = 1.85, e^95% CI^ = 1.40 – 2.46) were associated with a higher odds.

Regarding sedentary behaviour (Table 5), respondents who cited work (β = 31.57, 95% CI: 9.54 to 53.60) and limited accessibility to exercise facilities (β = 28.83, 95% CI: 3.06 to 54.60) were more likely to be sedentary, whereas those who reported safety concerns (β =54.73, 95% CI: -81.43 to -28.01) were less likely to be sedentary.

**Table 5:** Adjusted models for the association between barriers to physical activity and sedentary behavior

Sedentary behaviour ^#^		
Barriers to physical activity	β (95% CI)	*p*-value
Work	31.57 (9.54 to 53.60)	0.005
Limited Accessibility of gym or other exercise facilities (e.g., distance, hours open, availability)	28.83 (3.06 to 54.60)	0.028
Safety concerns (e.g., street lighting, traffic)	-54.73 (-81.43 to -28.01)	< 0.001

^#^ Adjusted for age, ethnicity, educational qualification, marital status, personal income, body mass index, chronic conditions, overall physical activity, the weather, safety concerns and limited accessibility

## Discussion

Identifying significant perceived barriers to physical activity allow policymakers to implement effective strategies that increase physical activity and reduce sedentary behaviour. Our results showed that the three most common perceived barriers were a lack of time, fatigue, and pollution. Moreover, the significant correlates of physical activity-related outcomes were a lack of footpaths, cycle lanes or parks, cost, safety concerns, pollution, weather, lack of time, age and fatigue. Sedentary behaviour was associated with work, limited accessibility to exercise facilities and safety concerns.

Lack of time and fatigue as barriers to physical activity were also prevalent in several studies. In a study by Uijtdewilligen et al. on park use for physical activity in Singapore, the top three reasons for not visiting parks were being too busy with their work or studies, being too tired or choosing to stay at home, and being concerned about the weather [27]. A study in the United States also found that the four most common barriers to physical activity were lack of time, fatigue, adequate exercise at work, and low motivation [43].

However, pollution may be a seasonal barrier that was overestimated in the survey. Singapore experiences haze from forest fires in neighbouring Indonesia. It is a seasonal problem between July and October when the monsoonal winds tend to blow the smoke to Singapore. In September 2019, the haze caused the air quality to reach an exceptionally unhealthy level for 11 days [44–46]. Consequently, the prevalence of pollution as a barrier might be overstated because respondents might recall this event that affected their daily activities then.

Our study showed that lacking footpaths, cycle lanes or parks was negatively associated with the level of transport-related physical activity, which is consistent with other studies. According to a qualitative study on the attitudes toward walking and cycling in Singapore, the participants knew the benefit of cycling and were enthusiastic about cycling [47]. However, some individuals were discouraged from cycling due to a lack of cycling paths and parking facilities [47]. As our study assessed perceived barriers to physical activity, this association can arise because individuals may be unaware of the existing infrastructure. This possibility is supported by the study from Uijtdewilligen et al., which found that their participants were unaware of parks near their homes [27].

The level of leisure-related physical activity was lower for individuals citing the weather as a barrier. Similarly, another study conducted among older individuals in six European countries showed that humidity and temperature were positively associated with physical activity [48]. The study by Uijtdewilligen et al. explained this association by revealing that the weather might demotivate individuals from exercising outdoors, especially when Singapore is typically humid and warm [27]. Our results also presented that weather was associated with higher odds of engaging in leisure-related physical activity. This finding suggests that individuals still exercise despite perceiving the weather as a barrier, though they may exercise less.

Besides the weather, lack of time and age were negatively associated with the level of leisure-related physical activity. Furthermore, age, cost and fatigue were related to lower odds of engaging in leisure-related physical activity. These findings corroborated with other studies. In a study on the process evaluation of park prescription intervention in Singapore, the participants explained that they lacked the time to exercise because they were too busy with work and domestic duties [26]. Some studies have also found that individuals who perceived age as a barrier might be discouraged from exercising because they viewed the ageing process as an obstacle to being physically active, such as the increased risk of injury and difficulty in learning complex exercises [27, 49].

Regarding the association between cost and the odds of engaging in leisure-related physical activity, two qualitative studies found that cost of gym membership and fitness classes was a barrier to physical activity [50, 51]. Specifically, the focus group discussion by Eyler et al. revealed that older Asian women reported cost to be a barrier to physical activity because they were frugal [51], which is consistent with our findings as frugality is considered a virtue in Singapore [52]. The negative association between fatigue and the odds of engaging in leisure-related physical activity can be explained by the study from Uijtdewilligen et al., which found that individuals might be too exhausted to exercise after returning from work and doing housework chores [27].

Work was positively associated with sedentary behaviour in our study. Several studies have examined the association between work commitment and sedentary behaviour. Wang et al. examined the sedentary behaviour of employees in a tertiary hospital in Singapore and revealed that the median sitting time was five hours/day [53]. Furthermore, the environment lacked the infrastructure to reduce sedentary behaviour at work [53]. Employees also misunderstood that decreasing physical inactivity was equivalent to reducing sedentary behaviour [53]. However, findings can be inconsistent across studies because sedentary behaviour was defined differently. Our study assessed sedentary behaviour using GPAQ, which asks respondents about the number of hours spent sitting or reclining in a day. Additionally, it provides examples of sedentary behaviour, which includes “sitting or reclining at work” and “at home”. However, the study by Salmon et al. omitted the time spent reclining or sitting during work and focused on leisure-time sedentary behaviour [17]. Hence, their findings showed that individuals who cited work commitment as a barrier were less likely to engage in sedentary behaviour [17], which is different from our findings.

Limited accessibility was also associated with sedentary behaviour. This finding contrasted with most quantitative studies, which revealed no statistically significant relationship [54, 55]. However, qualitative studies provided possible explanations for the positive association between limited accessibility and sedentary behaviour. Lim-Seto et al. found that individuals with poor access to exercise facilities were more likely to stay home, thus encouraging sedentary behaviour [56]. Ding et al. revealed that people staying in China’s rural areas were sedentary because they had to travel long distances to exercise facilities [57]. These findings suggest that inadequate access to exercise facilities may encourage physical inactivity and subsequently promote sedentary behaviour. However, this explanation was inconsistent with our results, since limited accessibility was not associated with all outcomes of physical activity. Hence, further studies are needed to understand why limited accessibility may influence sedentary behaviour.

Some external barriers, specifically the weather, safety concern, pollution and costs, were positively related to physical activity-related outcomes. Safety concern was also negatively associated with sedentary behaviour. While counterintuitive, few studies have similar findings [58, 59]. A study in the United States showed a similar association between perceived safety concerns and physical activity. It suggested that individuals who walked more were more conscious of their surroundings [59]. Likewise, our findings imply that physically active individuals may experience these external barriers more acutely. Non-sedentary individuals are also more likely to encounter safety issues while doing everyday activities. Our results for the association between pollution and physical activity might also differ from most studies as they measured pollution objectively. For example, a study in China revealed that a 10 μg/m^3^ increase in PM2.5 reduced moderate-to-vigorous physical activity by 2.2 min [60].

Based on our findings, motivating people to exercise can be a viable way to overcome internal barriers (lack of time, age, cost and fatigue) to physical activity. Other Singapore-based studies also found that individuals can be motivated by providing social support and emphasizing the benefits of exercise as well as the cost of physical inactivity [26, 27]. External barriers to physical activity (weather and lacking footpaths, cycle lanes or parks) can be overcome by educating people about the existing infrastructure that allows them to exercise in the shade and travel by walking or biking [27]. Sedentary behaviour may be improved by implementing workplace interventions at a population level. Local studies have proposed possible interventions, such as creating awareness of sedentary behaviour at work and changing the workplace environment, to reduce the amount of time spent sitting at work [53, 61]. Future studies could examine whether implementing these strategies is effective and cost-efficient in improving physical activity and sedentary behaviour.

Our study has several limitations. Firstly, the cross-sectional study design limits the ability to establish causality in the relationship between internal barriers and outcomes. Secondly, we could not achieve our target sample size of 3000 due to Covid-19 restrictions. Thirdly, although the questionnaire was assessed in terms of content validity, we did not evaluate the construct validity of the questionnaire. Lastly, the factors associated with physical activity and sedentary behaviour were related to the characteristics of a developed country. Hence, our findings have limited generalisability, especially for developing countries. Despite these limitations, this study is one of the few in Singapore to identify significant barriers to physical activity that policymakers can target for population-level intervention.

## Conclusion

According to our findings, the three most common barriers to physical activity were a lack of time, fatigue, and pollution in Singapore. Physical activity-related outcomes were negatively associated with a lack of pavement or parks, weather, lack of time, age, cost and fatigue. Work was positively correlated with sedentary behaviour. Physical activity can be improved in Singapore by motivating people (providing social support and emphasizing cost and benefits) and raising awareness of existing infrastructure. Moreover, workplace interventions that inform individuals on sedentary behaviour and reduce the time spent sitting can reduce sedentary behaviour.

## Supplementary information

**Additional file 1: Table S1.** Prevalence of perceived barriers to physical activity stratified by Sex and Age group. **Table S2.** Prevalence of perceived barriers to physical activity stratified by ethnicity.

## Data Availability

The datasets used during the current study are available from the corresponding author on reasonable request. The questionnaires utilized in the study can be provided by contacting the corresponding author.

## Abbreviations

AIC: Akaike Information Criteria
B: Regression coefficient
CAPI: Computer-assisted personal interviews
e^β^: Exponential form of regression coefficient
e^95% CI^: Exponential form of 95% confidence interval
GPAQ: Global Physical Activity Questionnaire
IQR: Interquartile range
IRRC: Institutional Research Review Committee
ITE: Institute of Technical Education
MET: Metabolic equivalent
N: Unweighted sample size
SGD: Singapore Dollar
95% CI: 95% Confidence interval
WHO: World Health Organisation

## References

[1] World Health Organization: WHO. Physical activity [Internet]. World Health Organization. 2020 [cited 2021 Nov 15]. Available from: https://www.who.int/news-room/fact-sheets/detail/physical-activity.

[2] Pietiläinen KH, Kaprio J, Borg P, Plasqui G, Yki-Järvinen H, Kujala UM (2008). Physical inactivity and obesity: a vicious circle. Obesity (Silver Spring).

[3] Haskell WL, Blair SN, Hill JO (2009). Physical activity: health outcomes and importance for public health policy. Prev Med (Baltim).

[4] González K, Fuentes J, Márquez JL (2017). Physical Inactivity, Sedentary Behavior and Chronic Diseases. Korean J Fam Med.

[5] Knight JA (2012). Physical inactivity associated diseases and disorders. Ann Clin Lab Sci.

[6] Ding D, Lawson KD, Kolbe-Alexander TL, Finkelstein EA, Katzmarzyk PT, van Mechelen W (2016). The economic burden of physical inactivity: a global analysis of major non-communicable diseases. Lancet.

[7] Sedentary Behaviour Research Network. Letter to the editor: standardized use of the terms “sedentary” and “sedentary behaviours”. Appl Physiol Nutr Metab. 2012;37(3):540–2. 10.1139/h2012-024 22540258

[8] Tremblay MS, Aubert S, Barnes JD, Saunders TJ, Carson V, Latimer-Cheung AE (2017). Sedentary Behavior Research Network (SBRN) – Terminology Consensus Project process and outcome. Int J Behav Nutr Phys Act.

[9] Müller AM, Chen B, Wang NX, Whitton C, Direito A, Petrunoff N (2020). Correlates of sedentary behaviour in Asian adults: A systematic review. Obes Rev.

[10] Mullane SL, Pereira MA, Buman MP, Leitzmann MF, Jochem C, Schmid D (2018). Sedentary Behaviour at the Community Level: Correlates, Theories, and Interventions. Sedentary Behaviour Epidemiology.

[11] van der Berg JD, Stehouwer CDA, Bosma H, van der Velde JHPM, Willems PJB, Savelberg HHCM (2016). Associations of total amount and patterns of sedentary behaviour with type 2 diabetes and the metabolic syndrome: The Maastricht Study. Diabetologia.

[12] Kim J, Im J-S, Choi Y-H (2017). Objectively measured sedentary behavior and moderate-to-vigorous physical activity on the health-related quality of life in US adults: The National Health and Nutrition Examination Survey 2003–2006. Qual life Res an Int J Qual life Asp Treat care Rehabil.

[13] Bowles HR, Morrow JRJ, Leonard BL, Hawkins M, Couzelis PM (2002). The association between physical activity behavior and commonly reported barriers in a worksite population. Res Q Exerc Sport.

[14] Herazo-Beltrán Y, Pinillos Y, Vidarte J, Crissien E, Suarez D, García R (2017). Predictors of perceived barriers to physical activity in the general adult population: a cross-sectional study. Brazilian J Phys Ther.

[15] Hoare E, Stavreski B, Jennings GL, Kingwell BA. Exploring Motivation and Barriers to Physical Activity among Active and Inactive Australian Adults. Sport (Basel, Switzerland). 2017;5(3):47. 10.3390/sports5030047 PMC5968958 29910407

[16] Al-Baho AK, Al-Naar A, Al-Shuaib H, Panicker JK, Gaber S (2016). Levels of Physical Activity among Kuwaiti Adults and Perceived Barriers. Open Public Health J.

[17] Salmon J, Crawford D, Owen N, Bauman A, Sallis JF (2003). Physical activity and sedentary behavior: A population-based study of barriers, enjoyment, and preference. Heal Psychol.

[18] Epstein LH, Roemmich JN, Saad FG, Handley EA (2004). The value of sedentary alternatives influences child physical activity choice. Int J Behav Med.

[19] Carlin A, Murphy MH, Gallagher AM (2015). Current influences and approaches to promote future physical activity in 11–13 year olds: a focus group study. BMC Public Health.

[20] Population in Brief 2020 [Internet]. Prime Minister’s Office. Singapore; 2020. Available from: https://www.strategygroup.gov.sg/files/media-centre/publications/population-in-brief-2020.pdf

[21] National Population Health Survey 2019 [Internet]. Singapore; 2019. Available from: https://www.moh.gov.sg/docs/librariesprovider5/default-document-library/nphs-2019-survey-report.pdf

[22] National Steps Challenge Community Challenge [Internet]. Healthhub.sg. [cited 2021 Nov 15]. Available from: https://www.healthhub.sg/programmes/124/community-challenge

[23] Yao J, Tan CS, Chen C, Tan J, Lim N, Müller-Riemenschneider F. Bright spots, physical activity investments that work: National Steps Challenge, Singapore: a nationwide mHealth physical activity programme. Br J Sports Med. 2020 Sep 1;54(17):1047 LP – 1048. 10.1136/bjsports-2019-101662 31857340

[24] Phan TP, Alkema L, Tai ES, Tan KHX, Yang Q, Lim W-Y (2014). Forecasting the burden of type 2 diabetes in Singapore using a demographic epidemiological model of Singapore. BMJ open diabetes Res care.

[25] Chan GC, Teo BW, Tay JC, Chen C-H, Cheng H-M, Wang T-D (2021). Hypertension in a multi-ethnic Asian population of Singapore. J Clin Hypertens.

[26] Petrunoff N, Yao J, Sia A, Ng A, Ramiah A, Wong M (2021). Activity in nature mediates a park prescription intervention’s effects on physical activity, park use and quality of life: a mixed-methods process evaluation. BMC Public Health.

[27] Uijtdewilligen L, Waters CNH, Aw S, Wong ML, Sia A, Ramiah A (2019). The Park prescription study: Development of a community-based physical activity intervention for a multi-ethnic Asian population. PLoS ONE.

[28] National Health Survillance Survey 2007 [Internet]. Singapore; 2007. Available from: https://www.moh.gov.sg/docs/librariesprovider5/resources-statistics/reports/nhss2007.pdf/

[29] AshaRani PV, Abdin E, Kumarasan R, Siva Kumar FD, Shafie S, Jeyagurunathan A (2020). Study protocol for a nationwide Knowledge, Attitudes and Practices (KAP) survey on diabetes in Singapore’s general population. BMJ Open.

[30] Wee HL, Ho HK, Li SC (2002). Public awareness of diabetes mellitus in Singapore. Singapore Med J.

[31] Pan SY, Cameron C, Desmeules M, Morrison H, Craig CL, Jiang X (2009). Individual, social, environmental, and physical environmental correlates with physical activity among Canadians: a cross-sectional study. BMC Public Health.

[32] Humpel N, Owen N, Leslie E (2002). Environmental factors associated with adults’ participation in physical activity: A review. Am J Prev Med.

[33] Kelly S, Martin S, Kuhn I, Cowan A, Brayne C, Lafortune L (2016). Barriers and Facilitators to the Uptake and Maintenance of Healthy Behaviours by People at Mid-Life: A Rapid Systematic Review. PLoS ONE.

[34] Armstrong T, Bull F (2006). Development of the World Health Organization Global Physical Activity Questionnaire (GPAQ). J Public Health (Bangkok).

[35] WHO. The WHO STEPwise approach to chronic disease risk factor surveillance. WHO STEPS Surveill Man. 2005;490.

[36] Chu AHY, Ng SHX, Koh D, Müller-Riemenschneider F (2015). Reliability and Validity of the Self- and Interviewer-Administered Versions of the Global Physical Activity Questionnaire (GPAQ). PLoS ONE.

[37] Chu AHY, Ng SHX, Koh D, Müller-Riemenschneider F (2018). Domain-Specific Adult Sedentary Behaviour Questionnaire (ASBQ) and the GPAQ Single-Item Question: A Reliability and Validity Study in an Asian Population. Int J Environ Res Public Health.

[38] Lau JH, Nair A, Abdin E, Kumarasan R, Wang P, Devi F (2021). Prevalence and patterns of physical activity, sedentary behaviour, and their association with health-related quality of life within a multi-ethnic Asian population. BMC Public Health.

[39] O’Donoghue G, Perchoux C, Mensah K, Lakerveld J, Van Der Ploeg H, Bernaards C, et al. A systematic review of correlates of sedentary behaviour in adults aged 18–65 years: A socio-ecological approach. BMC Public Health. 2016;16:163. 10.1186/s12889-016-2841-3 PMC4756464 26887323

[40] Bauman AE, Reis RS, Sallis JF, Wells JC, Loos RJF, Martin BW (2012). Correlates of physical activity: Why are some people physically active and others not?. Lancet.

[41] Slymen DJ, Ayala GX, Arredondo EM, Elder JP (2006). A demonstration of modeling count data with an application to physical activity. Epidemiol Perspect Innov.

[42] Cerin E, Leslie E, Sugiyama T, Owen N (2010). Perceived barriers to leisure-time physical activity in adults: an ecological perspective. J Phys Act Health.

[43] Brownson RC, Baker EA, Housemann RA, Brennan LK, Bacak SJ (2001). Environmental and policy determinants of physical activity in the United States. Am J Public Health.

[44] Haze hits unhealthy levels across Singapore on Wednesday. Channel Newsasia [Internet]. 2019 Sep 18; Available from: 2019

[45] Prisca A. Haze in S’pore: Air quality hits unhealthy level again, could worsen on Formula 1 race day. Straits Times. 2019 Sep 21;

[46] Climate and Air Quality [Internet]. Department of Statistics Singapore. 2021 [cited 2021 Dec 23]. Available from: https://www.singstat.gov.sg/publications/reference/ebook/society/climate-and-air-quality

[47] Rojas López MC, Wong YD (2017). Attitudes towards active mobility in Singapore: A qualitative study. Case Stud Transp Policy.

[48] Timmermans EJ, van der Pas S, Dennison EM, Maggi S, Peter R, Castell MV, et al. The Influence of Weather Conditions on Outdoor Physical Activity Among Older People With and Without Osteoarthritis in 6 European Countries. J Phys Act Health. 2016/08/24. 2016 Dec;13(12):1385–95. 10.1123/jpah.2016-0040 PMC5384632 27633622

[49] Crombie IK, Irvine L, Williams B, McGinnis AR, Slane PW, Alder EM (2004). Why older people do not participate in leisure time physical activity: a survey of activity levels, beliefs and deterrents. Age Ageing.

[50] Clark DO (1999). Identifying Psychological, Physiological, and Environmental Barriers and Facilitators to Exercise Among Older Low Income Adults. J Clin Geropsychology.

[51] Eyler AA, Baker E, Cromer L, King AC, Brownson RC, Donatelle RJ (1998). Physical activity and minority women: a qualitative study. Heal Educ Behav Off Publ Soc Public Heal Educ.

[52] Tan C, Tan CS (2014). Fostering Social Cohesion and Cultural Sustainability: Character and Citizenship Education in Singapore. Diaspora, Indig Minor Educ.

[53] Wang NX, Chen J, Wagner NL, Rebello SA, Petrunoff NA, Owen N (2020). Understanding and Influencing Occupational Sedentary Behavior: A Mixed-Methods Approach in a Multiethnic Asian Population. Heal Educ Behav.

[54] Wallmann-Sperlich B, Bucksch J, Hansen S, Schantz P, Froboese I (2013). Sitting time in Germany: an analysis of socio-demographic and environmental correlates. BMC Public Health.

[55] Fields R, Kaczynski AT, Bopp M, Fallon E (2013). Built environment associations with health behaviors among hispanics. J Phys Act Health.

[56] Tam-Seto L, Weir P, Dogra S (2016). Factors Influencing Sedentary Behaviour in Older Adults: An Ecological Approach. AIMS public Heal.

[57] Ding D, Sallis JF, Hovell MF, Du J, Zheng M, He H (2011). Physical activity and sedentary behaviours among rural adults in suixi, china: a cross-sectional study. Int J Behav Nutr Phys Act.

[58] Mertens L, Compernolle S, Gheysen F, Deforche B, Brug J, Mackenbach JD (2016). Perceived environmental correlates of cycling for transport among adults in five regions of Europe. Obes Rev.

[59] Carlson JA, Bracy NL, Sallis JF, Millstein RA, Saelens BE, Kerr J (2014). Sociodemographic moderators of relations of neighborhood safety to physical activity. Med Sci Sports Exerc.

[60] Yu M, Wu Y, Gordon SP, Cheng J, Chen P, Wang Y (2021). Objectively measured association between air pollution and physical activity, sedentary behavior in college students in Beijing. Environ Res.

[61] Waters CN, Ling EP, Chu AHY, Ng SHX, Chia A, Lim YW (2016). Assessing and understanding sedentary behaviour in office-based working adults: a mixed-method approach. BMC Public Health.

