# Supplementary material for: A cross-sectional study on the perceived barriers to physical activity and their associations with domain-specific physical activity and sedentary behaviour
Source: BMC Public Health. 2022 May 26;22:1051. doi: 10.1186/s12889-022-13431-2 (PMC9132675; doi:10.1186/s12889-022-13431-2)
Supplement: Supplementary file 1 — Additional file 1: Table S1. Prevalence of perceived barriers to physical activity stratified by Sex and Age group. Table S2. Prevalence of perceived barriers to physical activity stratified by ethnicity. [file 12889_2022_13431_MOESM1_ESM.docx]

Supplemental Table 1: Prevalence of perceived barriers to physical activity stratified by Sex and Age group

|  | Somewhat of a barrier/Very much a barrier | | | | | |
| --- | --- | --- | --- | --- | --- | --- |
|  | Sex | | Age group | | | |
|  | Female (*n* = 1458) | Male (*n* = 1409) | 18 -34 (*n* = 814) | 35 - 49 (*n* = 711) | 50 - 64 (*n* = 766) | 65 and above  (*n* = 576) |
|  | Weighted % (*n*) | Weighted % (*n*) | Weighted % (*n*) | Weighted % (*n*) | Weighted % (*n*) | Weighted % (*n*) |
| A disability or injury | 18.7% (309) | 20.5% (315) | 19.2% (174) | 16.2% (123) | 20.1% (172) | 25.8% (155) |
| Young children or family needs | 30.2% (448) | 22.3% (305) | 26.0% (229) | 37.8% (290) | 19.5% (168) | 17.9% (66) |
| Work | 46.6% (609) | 49.7% (651) | 63.3% (508) | 57.3% (381) | 39.6% (289) | 15.9% (82) |
| The weather (e.g., wet and hot) | 51.4% (686) | 47.7% (577) | 53.9% (417) | 54.9% (338) | 42.4% (304) | 44.1% (204) |
| Pollution - Haze | 58.0% (804) | 54.2% (699) | 54.8% (447) | 60.4% (395) | 55.0% (405) | 52.8% (256) |
| Lack of time | 66.4% (892) | 64.0% (862) | 80.7% (651) | 77.3% (520) | 53.4% (417) | 33.5% (166) |
| Cost | 24.8% (348) | 19.0% (295) | 27.7% (244) | 22.2% (169) | 17.9% (155) | 17.6% (75) |
| Safety concerns (e.g., street lighting, traffic) | 26.5% (367) | 19.8% (258) | 22.0% (191) | 24.0% (159) | 23.9% (176) | 22.9% (99) |
| Limited accessibility of gym or other exercise facilities (e.g., distance hours, open, availability) | 29.1% (376) | 22.5% (309) | 37.1% (275) | 26.5% (187) | 20.7% (165) | 11.5% (58) |
| Age | 19.0% (306) | 20.4% (278) | 5.8% (46) | 16.5% (112) | 27.8% (204) | 38.6% (222) |
| Lack of footpaths, cycle lanes or parks | 15.9% (219) | 16.0% (195) | 13.9% (123) | 15.0% (94) | 19.1% (126) | 16.4% (71) |
| Feeling tired | 66.4% (930) | 62.9% (830) | 71.1% (577) | 70.3% (448) | 60.2% (443) | 50.0% (292) |
| Abbreviation: *n* – unweighted sample size. | | | | | | |

Supplemental Table 2: Prevalence of perceived barriers to physical activity stratified by ethnicity

|  | Somewhat of a barrier/Very much a barrier | | | |
| --- | --- | --- | --- | --- |
|  | Ethnicity | | | |
|  | Chinese (*n* = 791) | Malay (*n* = 961) | Indian (*n* = 908) | Others (*n* = 207) |
|  | Weighted % (*n*) | Weighted % (*n*) | Weighted % (*n*) | Weighted % (*n*) |
| A disability or injury | 18.8% (151) | 23.9% (228) | 20.1% (201) | 20.2% (44) |
| Young children or family needs | 25.0% (195) | 30.9% (270) | 29.2% (236) | 33.0% (52) |
| Work | 48.1% (371) | 47.0% (398) | 47.1% (390) | 56.1% (101) |
| The weather (e.g., wet and hot) | 51.6% (407) | 44.8% (415) | 40.1% (348) | 47.3% (93) |
| Pollution - Haze | 57.1% (451) | 57.5% (532) | 46% (414) | 53.8% (106) |
| Lack of time | 65.1% (506) | 65.8% (580) | 63.3% (532) | 73.2% (136) |
| Cost | 20.9% (163) | 29.5% (264) | 20.5% (174) | 23.5% (42) |
| Safety concerns (e.g., street lighting, traffic) | 23.3% (185) | 27.7% (251) | 14.6% (135) | 27.6% (54) |
| Limited accessibility of gym or other exercise facilities (e.g., distance hours, open, availability) | 25.9% (200) | 28.2% (250) | 23.4% (192) | 22.2% (43) |
| Age | 20.3% (166) | 19.8% (218) | 13.5% (157) | 20.3% (43) |
| Lack of footpaths, cycle lanes or parks | 16.4% (131) | 15.8% (144) | 11.9% (109) | 17.0% (30) |
| Feeling tired | 65.7% (514) | 63.9% (588) | 59.3% (540) | 60.4% (118) |
| Abbreviation: *n* – unweighted sample size. | | | | |
